# MCAM/CD146 Signaling *via* PLCγ1 Leads to Activation of β_1_-Integrins in Memory T-Cells Resulting in Increased Brain Infiltration

**DOI:** 10.3389/fimmu.2020.599936

**Published:** 2020-12-14

**Authors:** Lisa Zondler, Sebastian Herich, Petra Kotte, Katharina Körner, Tilman Schneider-Hohendorf, Heinz Wiendl, Nicholas Schwab, Alexander Zarbock

**Affiliations:** ^1^ Department of Anesthesiology, Intensive Care and Pain Medicine, University of Münster, Münster, Germany; ^2^ Department of Neurology with Institute of Translational Neurology, University of Münster, Münster, Germany

**Keywords:** integrin, T-cell, adhesion, recruitment, multiple sclerosis, neuroinflammation, melanoma cell adhesion molecule/CD146, PLCγ1 activation

## Abstract

Multiple sclerosis is a chronic auto-inflammatory disease of the central nervous system affecting patients worldwide. Neuroinflammation in multiple sclerosis is mainly driven by peripheral immune cells which invade the central nervous system and cause neurodegenerative inflammation. To enter the target tissue, immune cells have to overcome the endothelium and transmigrate into the tissue. Numerous molecules mediate this process and, as they determine the tissue invasiveness of immune cells, display great therapeutic potential. Melanoma cell adhesion molecule (MCAM) is a membrane-anchored glycoprotein expressed by a subset of T-cells and MCAM+ T-cells have been shown to contribute to neuroinflammation in multiple sclerosis. The role of the MCAM molecule for brain invasion, however, remained largely unknown. In order to investigate the role of the MCAM molecule on T-cells, we used different *in vitro* and *in vivo* assays, including *ex vivo* flow chambers, biochemistry and microscopy experiments of the mouse brain. We demonstrate that MCAM directly mediates adhesion and that the engagement of MCAM induces intracellular signaling leading to β1-integrin activation on human T-cells. Furthermore, we show that MCAM engagement triggers the phosphorylation of PLCγ1 which is required for integrin activation and thus amplification of the cellular adhesive potential. To confirm the physiological relevance of our findings *in vivo*, we demonstrate that MCAM plays an important role in T-cell recruitment into the mouse brain. In conclusion, our data demonstrate that MCAM expressed on T-cells acts as an adhesion molecule and a signaling receptor that may trigger β1-integrin activation *via* PLCγ1 upon engagement.

## Introduction

Multiple sclerosis (MS) is a common autoimmune disease of the central nervous system (CNS) that is characterized by demyelination [reviewed in (1)]. One hallmark of MS is the wave-wise invasion of peripheral immune cells into the CNS forming so-called plaques which are characterized by high inflammatory activity, progressive destruction of the myelin sheath and neuronal death [reviewed in (1)]. Several previous studies have shown that, besides myeloid cells, blood-derived T-cells are the major drivers of local CNS inflammation in MS and among these, particularly T-cells of the Il-17 producing subpopulation (Th17/Tc17 subtype) (2–6). Tissue invasion of immune cells includes a highly regulated recruitment cascade that consists of tethering, adhesion, rolling and transmigration allowing the cells to leave the blood stream and to enter the target tissue. To overcome the blood brain barrier (BBB), T-cells largely rely on the interaction of the integrin VLA-4 (very late antigen-4; β1α4; CD49d/CD29) with endothelial VCAM-1 (vascular cell adhesion molecule 1) (7–9) and thus, Natalizumab, an antibody blocking VLA-4, is commonly used as a therapeutic approach (8). However, also VLA-4 independent mechanisms for T-cell recruitment into the brain must exist as patients experience relapses even during Natalizumab therapy (10).

Melanoma cell adhesion molecule (MCAM; CD146, MUC18) is an integral membrane glycoprotein of the immunoglobulin gene superfamily, which initially has been described in the context of cancer metastasis and tissue invasiveness of melanoma (11–13). MCAM is broadly expressed in embryonic mesenchymal tissue indicating important roles for embryonic development (14–20). However, its expression pattern in healthy adult tissue is very distinct and limited to rather small subsets of B- (21) and T-cells (22, 23), as well as the intercellular junctions of endothelial cells (24, 25). Both homophilic MCAM-MCAM interactions (26–28) and heterophilic interactions of MCAM with laminin-411 (15, 29–31) have been described. MCAM has been described as an adhesion receptor, but also to induce intracellular signaling in endothelial cells. Binding to endothelial MCAM induces protein kinase phosphorylation events resulting in Ca2+ burst and actin skeleton rearrangements (32, 33), supporting a role for MCAM in cell adhesion and motility.

MCAM expression has been linked to subsets of IL-17 producing T-cells (31, 34–39) and is in fact a criterium for the definition of Th17 cells (37, 40). We and others have previously shown that MCAM expression improves adherence of T-cells in an *in vitro* model of the BBB (41) and penetration of the blood cerebrospinal fluid barrier (BCSFB) *in vitro* and *in vivo* (23, 31) and further, that MCAM expressing T-cells reside to active lesion sites in MS patients (41). Thus, MCAM expression might be an important mechanism of CNS invasiveness of T-cells. As the particular function of MCAM in T-cell migration remains elusive so far, the aim of this study was to characterize the contribution of MCAM-ligand interactions to T-cell invasion into the CNS using primary human and murine MCAM expressing effector memory – and central memory T-cells (T_EM_/T_CM_) mechanistically by using different *in vitro* and *in vivo* approaches analyzing both adhesion and intracellular signaling.

## Materials and Methods

### Ethics Approval

All experiments including human material were approved by the local ethics committee (Ethik-Kommission der Ärztekammer Westfalen-Lippe und der Medizinischen Fakultät der Westfälischen-Wilhelms-Universität, registration number 2010-245-f-S) and performed according to the Declaration of Helsinki. All experiments involving mice were approved by the responsible animal protection authority (Landesamt für Natur- Umwelt- und Verbraucherschutz Nordrhein-Westfalen) and conducted according to the German Tierschutzgesetz.

### Mice

Spleens from 2D2 mice (male and female, 8–12 weeks of age) were used to isolate myelin oligodendrocyte glycoprotein-specific T-cells (kind gift from Luisa Klotz, Neurology Department, Münster) (23). C57BL/6J wild-type mice were purchased from Jackson Laboratory.

### Isolation and Fluorescence Activated Cell Sorting of Human MCAM+/- Effector and Central Memory T-Cells

CD4+ T cells were isolated from fresh human blood samples of healthy donors by density gradient centrifugation using Phosphate Buffered Saline (PBS) (Sigma), RosetteSep™ Human CD4+ T Cell Enrichment Cocktail (Stemcell Technologies Inc.), and Lymphoprep. Cells were cultivated in RPMI-1640 medium (PAN Biotech) supplemented with 10% heat-inactivated FCS (Sigma) and 1% Penicillin/Streptavidin (PAN Biotech). MCAM+/- CD45RA- CD62L+ central memory (T_CM_) and MCAM+/- CD45RA- CD62L- effector memory (T_EM_) cells were isolated using fluorescence activated cell (FAC) sorting with a FACSAria III Cell Sorter (BD Bioscience). For labeling of cell surface molecules and subsequent FAC sorting, CD4+ T cell subpopulations were stained with fluorochrome-conjugated antibodies targeting CD45RA, CD62L (both Biolegend), and CD146/MCAM (BD Bioscience) diluted in PBS + 0.5% BSA (Biomol) + 2 mM Ethylenediaminetetraacetic acid (Sigma) for 30 min at 4°C.

### Flow Cytometry of Human MCAM+/- Effector and Central Memory T-Cells

Cells were washed in PBS + 10% FCS and stained with primary antibodies (anti-CD45RA-BV421, anti-CD62L-APC-Cy7, anti CDCD49f-FITC, anti-CD51-APC, anti CD493-FITC, anti-CD49b-APC, anti-CD49a-APC, all Biolegend; anti-IL17a-Alexa647 and anti-CD146/MCAM-PE, both BD Bioscience) for 30 min on ice. Intracellular staining of IL-17a was performed by implementation of the CytoFAST Fix/Perm buffer set (Biolegend) exactly according to the manufacturer’s instructions. The cells were fixed in 4% PFA (Sigma) and the stainings were assessed on a BD Canto II flow cytometer.

### Treatment and Culture of Human Primary T-Cells

Pretreatments for flow chamber and VCAM-1 binding assays included kinase inhibitors and blocking antibodies. The cells were diluted to 1*10^6/ml in RPMI1640 (PAN Biotech) + 10% FCS (Sigma) + 1% PS (PAN Biotech) and incubated for 30 min at 37°C with SRC inhibitor pp2 or the respective control pp3 (both Sigma; final concentration 20 µM), the Plcγ inhibitor U73122 or the respective control U73433 (both Thermo; final concentration 5 µM) or the FAK1 inhibitor FAK14 (Invitrogen, final concentration 5 µM), Natalizumab/anti-VLA-4 (Tysabri; final concentration 10 µg/ml) or anti VLA-2 (Millipore; final concentration 10 µg/ml). MCAM blocking was performed using anti MCAM (clone2107, Prothena; final concentration 10 µg/ml).

### Flow Chamber Assays

Flow chamber assays were performed as described previously (41–45). Briefly, chambers were coated for 2 h at RT using recombinant human VCAM-1 (R&D; concentration as indicated in results section), recombinant human MCAM (Thermo; concentration as indicated in results section), and recombinant human laminin-411 (Biolamina, concentration as indicated in results section). Chambers were blocked in Casein (Blocker TM Casein; Thermo) for 1 h at RT. For the measurements, the cells were diluted to 1*10^6/ml in RPMI1640 (PAN Biotech) + 10% FCS (Sigma) + 1% PS (PAN Biotech), constant flow was established for 1 min and the flow was stopped for 30 s. Then constant flow was reestablished and then the adhering cells in the whole area of the capillary (0.02 x 0.2 mm) were counted under flow within 1 min. All flow chamber experiments were performed in a completely standardized way concerning the time of flow, the temperature (RT), the shear stress applied, the dilution of the cells and by one researcher blinded concerning the respective population and treatment. Regarding adherence to MCAM and laminin-411 all different conditions were assessed with cells from each donor to provide maximal comparability.

### Phalloidin Staining of Human MCAM+/- Central Memory T-Cells

Glass cover slips were coated with human recombinant MCAM (Thermo; 10 µg/ml)/laminin-411 (Biolamina; 10 µg/ml) for 2h at RT. The cells were diluted to 1*10^6/ml in RPMI1640 (PAN Biotech) + 10% FCS (Sigma) + 1% PS (PAN Biotech) and incubated rolling on the coated cover slips on a shaker for 1h at RT at 100 rpm. Then, 8% PFA (Sigma) in PBS was carefully added (final concentration 4% PFA) to the cells to fix them *in situ*. After incubating at RT for 10 min, the cells were permeabilized in PBS (PAN Biotech) + 100 mM Glycine (Sigma) + 0.1% triton (AppliChem) for 10 min at RT. Cells were stained in Alexa Fluor647 Phalloidin (Thermo; 1:40 in PBS + 1% BSA) for 30 min at RT in the dark. Next, the cells were carefully washed in PBS and stained with DAPI (Sigma; 1µg/ml in PBS) for 10 min at RT in the dark. Cells were washed in PBS and mounted on object slides using fluorescence mounting medium (Dako).

### *In Vitro* Stimulation by Rolling

Cell culture plates were coated with human recombinant MCAM (Thermo; 10 µg/ml)/laminin-411 (Biolamina; 10 µg/ml) for 2 h at RT. The cells were diluted to 1*10^6/ml in RPMI1640 (PAN Biotech) + 10% FCS (Sigma) + 1% PS (PAN Biotech) and incubated rolling on the coated plates on a shaker for 1 h at RT at 100 rpm, harvested in 100 µl Rap/Rac lysis buffer (50 mM Tris PH = 7.4, 500 mM NaCl, 25 mM NaF, 2.5 mM MgCl2, 1 mM NaOrthovanadate, 10% glycerol, 1% NP40; all Sigma) complemented with HALT protease cocktail (1:100; Thermo).

### VCAM-1 Binding Assays

Cells were diluted to 2*10^6/ml. 0,2 µg recombinant human VCAM-1 (R&D) per sample was labelled by adding anti human Fc- antibody (APC, Southern Biotech; 5:1) on ice for 10 min. Cells were stimulated by rolling over MCAM ligands as described above. Labelled VCAM-1 was added to the cells and incubated for 30 min at 37°C, cells were fixed in 4% PFA (Sigma) and VCAM binding was assessed on a BD Canto II flow cytometer.

### Electroporation of Human T-Cells

Electroporation of human T-cells was performed using the Amaxa human T-Cell Nucleofector Kit (Lonza, VPA-1002) and the Nucleofactor 2b system (Lonza), according to the manufacturer’s instructions using RPMI1640 (PAN Biotech) + 20% FCS (Sigma) + 1% PS (PAN Biotech). The cells were diluted to 1*10^6/ml. Centrifugation steps were performed at 400 x g for 5 min and the cells were incubated for 30 min at 37°C + 5% CO_2_ after electroporation before plating. For the PLCγ1 knock down predesigned silencer siRNA (nc and B 100 nM; ABC 300 nM; Human siRNA Oligo Duplex, locus ID 5335, origene) were used.

### FACS-Based β1-Integrin Activation Assay

Cells were harvested by adding 500 µl PBS (PAN Biotech), centrifuged, devided into +/- MnCl_2_ samples and stained with anti-human β1-integrin open conformation specific Antibody (1:50, CHEMICON) for 30 min on ice. MnCl_2_ (1 µM; Sigma) was added to the respective samples as positive control. Next, the stained cells were washed twice in PBS and stained with anti-mouse secondary antibody (1:100; donkey anti mouse-Alexa488, Cell Signaling) for 30 min on ice, washed twice in PBS and fixed in 4% PFA (Sigma). The staining was assessed on a BD Canto II flow cytometer.

### Western Blotting

Western blotting was performed using the BioRad system according to standard protocols. Cell lysates were diluted in 5x Lämmli buffer (1M Tris PH = 6.8; 10% SDS; 50% glycerol; 500 mM b-mercaptoethanol; 0.5% bromophenol blue; all Sigma), boiled for 10 min at 95°C and separated in 10–12% glycine gels, blotted on Amersham protran nitrocellulose membranes (pore size 0,45 µm; Sigma) and blocked in TBST + BSA (3%, PAN Biotech) for 1h at RT. Then the membranes were incubated with the following primary and secondary antibodies over night at 4°C or for 1h at RT, respectively: (rabbit anti SRCp416, 1:5,000; rabbit anti FAK1p397, 1:1,000; rabbit anti Plcγ1p783, 1:1,000; rabbit anti p38, 1:2,000; anti rabbit-HRP, 1:2,000; all Cell Signaling). Membranes weredeveloped using the ECL kit (GE Healthcare) and light sensitive Amersham Hyperfilms (GE Healthcare). Quantification of protein expression was performed by ImageJ. The bands were labelled as region of interest, background subtraction and area under the curve quantification was performed using the plot lanes and label peak option for gels and normalized to the expression of p38 on the same membrane for individual blots.

### Murine Cell Culture + Differentiation

T-cell differentiation was performed as described previously (23, 31). Briefly, 6 murine spleens were mashed and the resulting leukocytes incubated in RPMI1640 (PAN Biotech) + heat inactivated FCS (10%; Sigma), Penicillin/Streptomycin (1%; PAN Biotech), L-glutamine (1%; PAN Biotech), b-Mercaptoethanol (50 µM; Sigma), anti IFNg (5 µg/ml; ebioscience, clone XMG1.2), anti IL-4 (0.5 µg/ml; ebioscience, clone 11B11), human TGFb (5 ng/ml; R&D), murine IL-23 (20 ng/ml; R&D) on plates coated with anti CD3 (5 µg/ml; ebioscience) and anti CD28 (2.5 µg/ml; ebioscience). After 5 days of differentiation at 37°C + 5% CO_2_ CD4+ T-cells were isolated using the CD4+ T Cell Isolation Kit (Miltenyi Biotec) strictly according to the manufacturer’s instructions.

### Flow Cytometry of Murine T-Cells

Cells were washed in PBS (PAN Biotech) + 10% FCS (Sigma) and stained with primary antibodies (anti-CD4-FITC, anti-MCAM/CD146-PE-Cy7, anti-IL17a-Alexa647; all Biolegend; 1:100) for 30 min on ice. Intracellular staining of IL-17a was performed by implementation of the CytoFAST Fix/Perm buffer set (Biolegend) exactly according to the manufacturer’s instructions. The cells were fixed in 4% PFA (Sigma) and the stainings were assessed on a BD Canto II flow cytometer.

### Electroporation of Murine T-Cells

Electroporation of murine T-cells was performed using the Amaxa murine T-Cell Nucleofector Kit (Lonza) and the Nucleofactor 2b system (Lonza), according to the manufacturer’s instructions. Centrifugation steps were performed at 400 x g for 5 min and the cells were incubated for 30 min at 37°C + 5% CO_2_ after electroporation before plating. To knock down PLCγ1 in murine cells predesigned silencer siRNA (nc and A 300 nM, ABC 300 nM; Mouse siRNA Oligo Duplex, locus ID 18803, origene) were used.

### Adoptive Transfer of Murine T-Cells

Murine CD4+ T-cells were isolated from spleens of 6 2D2 transgenic mice (male + female, 8–12 weeks of age), differentiated and transfected as described above. 24 h post siRNA transfection, labelling with cell tracker green (CMFDA; Thermo) was performed according to the manufacturer’s instructions. The cells were washed and 3*10^6 T-cells in 100 µl NaCl were transplanted per recipient mouse by i.v. injection.

### Immunostaining of Choroid Plexus Explants

The choroid plexus was explanted 48 h post adoptive cell transfer from the fourth ventricle and transferred on glass object slides and incubated in PBS (PAN Biotech) + tween20 (0.3%; Sigma) at RT for 5 min, washed twice in PBS (PAN Biotech) for 5 min and fixed in PBS (PAN Biotech) + PFA (2,2%; Sigma), glucose (2%, Sigma), sodium acide (0.02%; Sigma) for 20 min at RT. Choroid plexus were then rinsed in PBS (PAN Biotech), fixed in methanol (100%, Sigma) for 6 min at RT, washed twice in PBS for 5 min and blocked in PBS (PAN Biotech) + BSA (1%; PAN Biotech), tween20 (0.3%; Sigma), normal goat serum (10%; Sigma) for 30 min at RT. The staining was performed in PBS (PAN Biotech) + tween20 (0.3%; Sigma) + primary antibody for 2h at RT (rat anti CD31; 1:100; BD). After washing the tissue twice in PBS (PAN Biotech) for 5 min, secondary stainings were performed using anti rat secondary antibodies (donkey anti rat-Alexa647; life technologies). Finally, the tissue was stained with DAPI (1 µg/ml; Sigma) and embedded in fluorescent mounting media (Dako).

### Immunohistochemistry

Brains were prepared 48 h post adoptive cell transfer. Brains were embedded in Tissue-Tek (Sakura) immediately after explantation and cut into 100 µm thick coronal tissue slices and stored at -80°C. Three sections per brain in 500 µm distance were stained and analyzed as follows. Sections were dried at RT for 30 min, fixed in ethanol-acetone (1:1, Sigma) for 10 min at RT, washed 3 times 5 min in PBS (PAN Biotech), blocked in PBS (PAN Biotech) + BSA (1%; PAN Biotech) + goat serum (10%; Sigma)+ tween20 (0.2%; Sigma) for 50 min at RT. Then sections were stained for 120 min at RT in PBS + BSA + goat serum + tween20 + primary antibody (rat anti CD31, BD; 1:100), washed 3 times 5 min in PBS and stained in PBS + 1% BSA + 10% goat serum + 0.2% tween20 + secondary antibody (donkey anti rat-Alexa647, life technologies; 1:100) for 60 min at RT in the dark. After washing 3 times in PBS (PAN Biotech), DAPI staining (1 µg/ml; Sigma) was performed for 10 at RT and the sections were embedded in fluorescent mounting media (Dako).

### Viability Test of Murine and Human T-Cells

For testing cell viability murine and human T-cells, a combination of the Zombie green fixable viability kit (Biolegend) and 7-AAD (Biolegend) was applied strictly according to the manufacturer’s instructions. Briefly, cells were washed in PBS (PAN Biotech) and resuspended in 100 µl PBS (PAN Biotech) + Zombie stain (1:500) per 1*10^6 cells, incubated for 30 min at RT in the dark and washed in PBS (PAN Biotech) + 10% FCS (Sigma). Then 7-AAD staining was performed 1:20 in PBS (PAN Biotech), the cells were fixed in 4% PFA (Sigma) and the cell viability was assessed on a BD Canto II flow cytometer.

### Microscopy

Microscopy was performed on a LSM700 system (Axio Observer.Z1; Zeiss) equipped with a HXP120c + LSM T-PMT lighting unit and Leica objectives (10x, 20x, 40x were used).

### Statistics

The indicated numbers of performed experiments (n) reflect the number of individual donors. Statistical analysis was performed using the Graphpad PRISM 6.01 software. For all experiments the distribution of values was assessed and accordingly, group comparisons were performed applying the appropriate T-test (normal or Mann-Whitney). Multiple comparisons were performed applying the two-way ANOVA including correction for multiple testing (Sidak). Statistical significance was assumed for p < 0.05 (*), p < 0.01 (**), p < 0.001 (***).

## Results

### Co-Coating of Flow Chambers With VCAM-1 and the MCAM Ligands MCAM and Laminin-411 Significantly Improves Adherence of MCAM+ Memory T-Cells

In all experiments, human central- and effector memory T-cells (T_CM_, T_EM_) were isolated by flourescence activated cell sorting (FACS) based on expression of CD4, CD45RA, and CD62L (T_CM_ CD4+CD45RA-CD62L+; T_EM_ CD4+ CD45RA-CD62L-) (**Figure S1 a**) (46). MCAM+ T_EM_ and T_CM_ thereby exhibited equal levels of MCAM expression (**Figure S1 b**) and expression of IL-17a was confirmed to be associated with the MCAM+ subpopulations of T_CM_ and T_EM_ (**Figure S1 c**).

**Figure 1:**
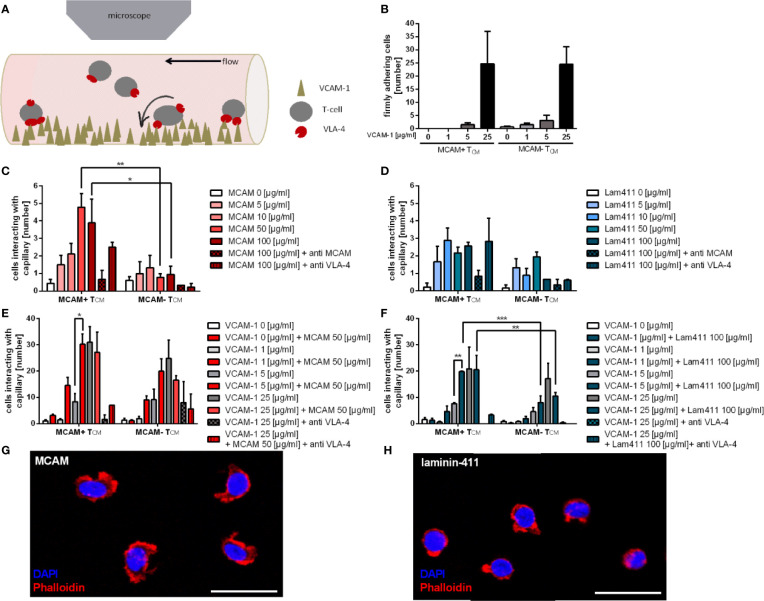
Co-coating of flow chambers with VCAM-1 and the MCAM ligands MCAM and laminin-411 significantly improves adherence of MCAM+ memory T-cells. Schematic presentation of the flow chamber assay illustrating how T-cells interact with the molecules coated on the capillary surface **(A)**. Firm adherence of MCAM+ and MCAM- T_CM_ was assessed to be dependent on the substrate dose in flow chambers coated with increasing amounts of VCAM-1 (0; 1; 5; 25 µg/ml; n = 6; **B**), MCAM (0; 5; 10, 50, 100 µg/ml; n = 3; **C**), and laminin-411 (0; 5; 10, 50, 100 µg/ml; n = 3; **D**) and regarding adherence to MCAM, also dependent on MCAM expression (50 µg/ml, p = 0.0039; 100 µg/ml, p = 0.0268; **C**). Firm adherence of MCAM+ and MCAM- T_CM_ was assessed in flow chambers using increasing amounts of VCAM-1 (0; 1; 5; 25 µg/ml) plus constant amounts of MCAM (50 µg/ml; p = 0.01; n = 3; **E**) or laminin-411 (100 µg/ml; p = 0.002; n = 3; **F**). Adherence was found to be increased in MCAM+ T_CM_ if VCAM-1 at 5 µg/ml was co-coated with MCAM or laminin-411 **(E, F)**, and compared to MCAM- T_CM_ in laminin-411 coated chambers (p < 0.001, p = 0.0093, **(F)**. Phalloidin stainings confirmed adherence of MCAM+ T_CM_ to MCAM **(G)** and laminin-411 **(H)**. Scale bars are 20 µm.

First, to analyze whether and how MCAM expression on different T-cell subsets affects the adhesive properties of human central- and effector memory T-cells (T_CM_, T_EM_), we performed flow chamber assays *in vitro* using primary human MCAM+ and MCAM- T_EM_ and T_CM_ separately (**Figure 1A**). However, as our assays analyzing MCAM mediated adhesion revealed no differences between T_EM_ and T_CM_ we focus on T_CM_ in the main figures and the data concerning T_EM_ is presented in the supplemental material. To provide a mechanistic analysis of adhesion to different substrates, all flow chamber assays were performed in a systematic way including standardized dilution of cells, shear and area of analysis.

To investigate whether the different T-cell subpopulations adhere to VCAM-1, we first used capillaries coated with different concentrations of VCAM-1 alone. The analyzed MCAM +/- T-cell populations demonstrated a dose dependent and comparable frequency of firm adherence (**Figure 1B**), thereby confirming the feasibility of the assay (7–9).

Both MCAM and laminin-411 are shown to be expressed on the endothelium or in the extracellular matrix (ECM) of the BBB as well as the choroid plexus (23, 31), which represent the main entry site of peripheral immune cells to the CNS (47, 48). As the physiological expression levels of endothelial MCAM have been reported to be variable (49, 50) we analyzed the adhesive properties of T-cellular MCAM by performing titration experiments using flow chambers coated with increasing amounts of MCAM (homophilic interaction) or laminin-411 (heterophilic interaction) using cells from the same donor to provide maximal comparability. We found that only MCAM expressing memory T-cells adhered to MCAM in our flow chambers (**Figure 1C**, **Figure S2 a**). In contrast, both MCAM+ and MCAM- T_CM_ adhered to laminin-411 **(** **Figure 1D**, **Figure S2 b**), probably due to the expression of laminin binding integrin α6 (**Figure S1 d/e**). The interaction of MCAM with both ligands was concentration dependent and specific, as the adhesion could be abolished by a blocking MCAM antibody **(** **Figures 1C, D**, **Figure S2 a/b**). In addition, we hypothesized that MCAM engagement induces an intracellular signaling cascade that leads to β1-integrin activation and thereby strengthens adhesion. To test this hypothesis, we used co-coated flow chambers with either MCAM or laminin-411 in combination with increasing concentrations of VCAM-1. We demonstrated that the adherence of MCAM+ T-cells is significantly increased in capillaries coated with MCAM/laminin-411 in addition to low concentrations of VCAM-1 (**Figures 1E, F**; **Figure S2 c/d**). This phenomenon exceeded the simple additive effect, thus indicating that MCAM binding indeed induces a signaling cascade supporting VLA-4 activation and VCAM-1 binding. This assumption was further affirmed by the fact that increased adherence to VCAM-1 in presence of laminin-411 was only induced in MCAM+ T_CM_, even though MCAM- T_CM_ also bind laminin-411 (**Figure 1F**). Morphological analyses of MCAM+ T_CM_ after adherence to MCAM/laminin-411 additionally point towards an activated phenotype upon interaction with the substrate (**Figures 1G, H**).

Control experiments further demonstrated MCAM-mediated adhesion to MCAM/laminin-411 + VCAM-1 to be VLA-4 but not VLA-2 dependent (**Figures S2 e–l**).

### Signaling *via* the MCAM Molecule Triggers SRC, FAK1 and PLCγ1 Activation in MCAM+ Memory T-Cells

**Figure 2:**
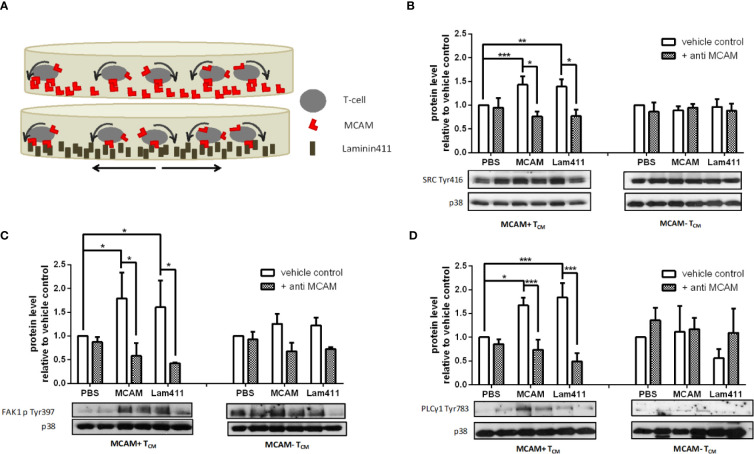
Signaling *via* the MCAM molecule triggers SRC, FAK1, and PLCγ1 activation in MCAM+ memory T-cells. *In vitro* rolling experiments in MCAM and laminin-411 coated dishes were performed to trigger MCAM signaling in T_CM_ as schematically represented in **(A)**. Western blotting analysis and quantification show increased phosphorylation of SRCp416 (**B**; p = 0.0.0006, p = 0.0029; n = 4), FAK1p397 (**C**, p = 0.01, p = 0.02; n = 3), and PLCγ1p783 (**D**, p = 0.01, p = 0.0006; n = 5) in MCAM+ T_CM_ upon interaction with the substrate. This effect is abolished by application of anti-MCAM antibody to the cells before plating them on the coated surface (**B**, p = 0.03, p = 0.02; **C**, p = 0.06, p = 0.07; **D**, p < 0.001, p < 0.001). Representative blots are shown for each kinase.

To investigate which signaling pathway is induced by MCAM engagement, we stimulated MCAM+ and MCAM- primary human memory T-cells with either MCAM or laminin-411 under shear conditions *in vitro* (**Figure 2A**). Induction of phosphorylation of the intracellular kinases SRC, FAK1 and PLCγ1 upon interaction with MCAM-specific ligands was only detectable in MCAM expressing T-cells, but not in MCAM- T-cells (**Figures 2B–D**, **Figures S3 a–f**), whereas PYK2, PLCγ2 or WASP did not get activated upon stimulation with MCAM-ligands (**Figures S3 g–j**). Importantly, increased phosphorylation of SRC, FAK1 and PLCγ1 was abolished in MCAM expressing T-cells pre-treated with a blocking MCAM antibody before stimulation (**Figures 2B–D**, **Figures S3 a–f**).

### Pharmacological Inhibition and Specific Knockdown of PLCγ1 Significantly Decreases VCAM-1 Binding in Primary Human MCAM+ Memory T-Cells by Inhibiting β1-Integrin Activation

**Figure 3:**
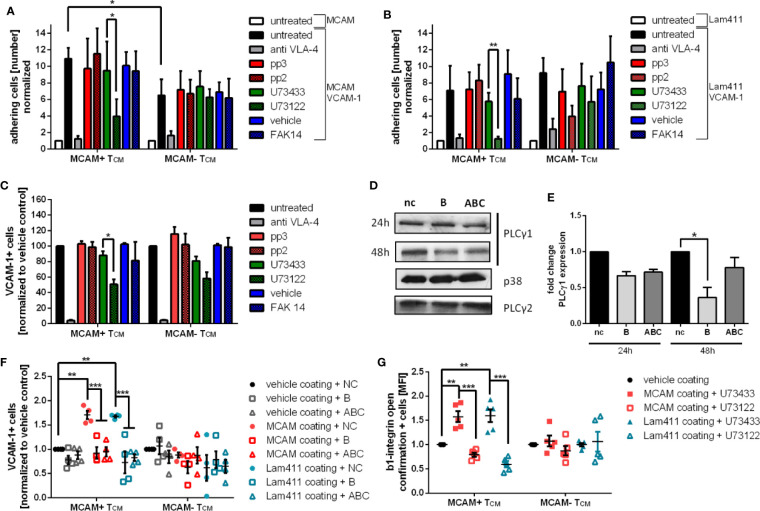
Pharmacological inhibition and specific knockdown of PLCγ1 significantly decreases VCAM-1 binding in primary human MCAM+ memory T-cells by inhibiting β1-integrin activation. To confirm the involvement of SRC, PLCγ and FAK1 in MCAM mediated intracellular signaling primary human T_CM_ were pretreated with the SRC inhibitor pp2 or the respective control pp3, the PLγ inhibitor U73122 or the respective control U73433, or the FAK1 inhibitor FAK14 before subjecting the cells to flow chamber assays. Flow chambers were coated with MCAM (50 µg/ml) or laminin-411 (100 µg/ml) plus VCAM-1 (5 µg/ml). Only inhibition of PLCγ by U73122 resulted in decreased adherence on MCAM + VCAM-1 or laminin-411 + VCAM-1 coated flow chambers in MCAM+ T_CM_ (**A**, p = 0.04, p = 0.0277, n = 4; **B**, p = 0.006, n = 4). These findings were confirmed in a VCAM-1 binding assay (**C**, p = 0.03, n = 5). To specify the inhibition of PLCγ to the subtype PLCγ1, a PLCγ1 knock down using specific siRNA oligonucleotides in primary human MCAM+ T cells was performed before analyzing the cells in MCAM/laminin-411 + VCAM-1 binding assays (nc, negative control; B, oligonucleotide B; ABC, mixture of oligonucleotide A, B, and C), representative western blots are shown in **(D)** and the quantification of the knock down in **(E)** (48 h, siRNA B p = 0.02, siRNA ABC p = 0.06). VCAM-1 binding assays using PLCγ1 knock down cells (48 h) further confirmed the induction of VCAM-1 binding upon MCAM stimulation with MCAM and laminin-411 protein as well as the involvement of PLCγ1 in MCAM mediated VCAM-1 binding (**F**, VCAM binding MCAM coating nc p = 0.0016, VCAM binding Laminin411 coating nc p = 0.003, MCAM coating siRNA B p = 0.0003, siRNA ABC p = 0.0006; Laminin coating siRNA B p < 0.0001, siRNA ABC p < 0.0001; n=4). Both MCAM stimulation with MCAM or laminin-411 results in increased abundance of full open β1-integrins on MCAM+ T_CM_ and can be reversed by administration of the PLCγ inhibitor U43122 as assessed by flow cytometry (**G**, MCAM coating U73433 p = 0.0038, U73122 p < 0.001; Laminin411 coating U73433 p = 0.0026, U73122 p < 0.001; n = 5).

To further substantiate the finding that MCAM signaling induces SRC, FAK1 and PLCγ1 activity, we blocked the activation of the different kinases by commercially available inhibitors. In flow chamber experiments using capillaries co-coated with VCAM-1 in combination with either MCAM or laminin-411, application of the PLCγ inhibitor U73122 significantly reduced adhesion compared to its control U73433 (**Figures 3A, B**, **Figure S4 a**). Notably, PLCγ inhibition only lead to decreased adherence in MCAM expressing memory T-cells, but not in MCAM- T-cells. These findings were further confirmed in a VCAM-1 binding assay (**Figure 3C** and **Figure S4 b**). Interestingly, in the VCAM-1 binding assays PLCγ inhibition lead to reduced VCAM-1 binding also in MCAM- T-cells suggesting that PLCγ plays a central role in VLA-4 activation.

As U73122 is not specific for PLCγ1 but also inhibits PLCγ2, we next sought to specify our findings by performing a specific PLCγ1 knock down in primary human memory T-cells using different siRNA oligonucleotides (**Figures 3D, E**; nc: negative control, B: siRNA B, ABC: mixture of siRNA A, B, and C). Cell viability was assessed by a flow cytometry-based approach and shown to be comparable in all treatment groups before and after the PLCγ1 knock down (**Figure S4 d**). VCAM-1 binding assays of oligonucleotide treated MCAM+/- memory T-cells were performed 48h after inducing the PLCγ1 knock down and confirmed that VCAM-1 binding was increased in MCAM+ T-cells upon *in vitro* stimulation with MCAM and laminin-411 (negative control). Importantly, this effect was absent in PLCγ1 knock down cells (oligonucleotides B, ABC) and in MCAM- T-cells (**Figure 3F**, **Figure S4 e**).

Next, we sought to go one step further and test whether we can additionally link MCAM signaling *via* PLCγ1 to β1-integrin activation. To that end, we used a flow cytometry-based assay to investigate β1-integrin activation (using an antibody that specifically detects the full active conformation) after *in vitro* stimulation of the cells with either MCAM or laminin-411. Here, we demonstrated that the stimulation with either MCAM or laminin-411 significantly increased β1-integrin activation in MCAM+ memory T-cells, but not in MCAM- cells (U73433; **Figure 3G** and **Figure S4 f**). Pharmacological inhibition of PLCγ also inhibited MCAM-triggered β1-integrin activation on MCAM+ memory T cells (U73122; **Figure 3G** and **Figure S4 f/g**).

### Knockdown of Plcγ1 Results in Impaired CNS Infiltration of MCAM+ T-Cells *In Vivo*

**Figure 4:**
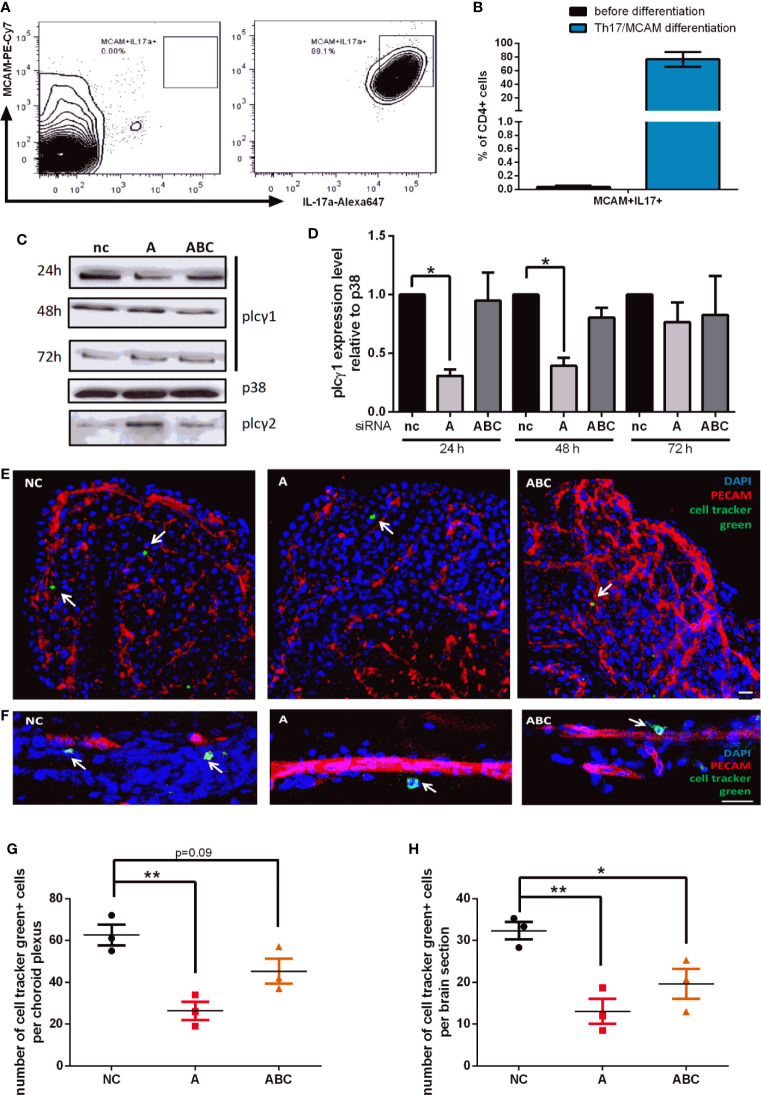
Knock down of Plcγ1 results in impaired CNS infiltration of MCAM+ T-cells *in vivo*. MCAM expressing murine T-cells were prepared from 2D2 mice, expanded and differentiated *in vitro*, representative data is shown in **(A, B)** (n = 3) and plcγ1 expression was knocked down by electroporation and transfer of specific siRNA oligonucleotides (nc, negative control; A, oligonucleotide A; ABC, mixture of oligonucleotide A, B, and C). Knock down efficiency was confirmed by western blotting **(C)** and quantified (**D**; siRNA A 24 h p = 0.02, 48 h p = 0.02; siRNA ABC 48 h p = 0.06). The abundance of MCAM+ plcγ1 knock down T-cells was then analyzed in cortex and choroid plexus by immunohistochemistry 48 h after adoptive transfer into wild-type mice as illustrated in representative images (**E**, choroid plexus; **F**, cortex). The number of transferred MCAM+ T-cells with plcγ1 knock down was significantly reduced both in choroid plexus (**G**, p = 0.005, p = 0.09; n = 3) and cortex (**H**, p = 0.006, p = 0.03; n = 3) in dependence of the knock down efficiency of the respective siRNA (nc, negative control; A, siRNA A; ABC, mixture of siRNA A, B, and C). Transplanted T-cells were stained with cell tracker green prior to transplantation (CMFDA, green), endothelium was stained using anti PECAM (Alexa647, red) and DAPI staining indicates cell nuclei (blue). Scale bar = 20 µm.

To analyze whether our finding that MCAM-triggered signaling leads to β1-integrin activation *via* PLCγ1 also affects T-cell behavior *in vivo*, we performed adoptive cell transfer experiments. For this, we first isolated CD4+ T-cells from spleens of 2D2 transgenic mice and cultured them under conditions that were reported to expand, activate and enrich MCAM+/Th17-cells (23, 31). On average 75% of CD4+ cells were shown to be MCAM+ IL17a+ after differentiation (**Figures 4A, B** **)**. Next, Plcγ1 knock down was performed using a set of different siRNAs. The knock down efficiency was variable between the different siRNAs as shown in **Figures 4C, D** **(**nc: negative control, A: siRNA A, ABC: mixture of siRNA A, B, and C) but cell viability was comparable between the differently treated cells (**Figure S5**). Twenty-four hours after inducing the Plcγ1 knock down the cells were labelled with cell tracker green, and re-transplanted into wildtype mice by i.v. injection. Forty-eight hours after injecting the cells, we analyzed the number of transplanted cells in the cortex and the plexus choroideus (4^th^ ventricle; **Figures 4E–H**) and found that knocking down Plcγ1 in MCAM+ Th17-cells significantly decreased the number of recruited cells in both analyzed compartments (**Figures 4G, H** **)** as illustrated in representative images [**Figure 4E** (choroid plexus), **F** (cortex)]. Importantly, the cell numbers in CNS tissue in our experiments were comparable to previous studies (23). These findings show that Plcγ1 signaling in MCAM+ T-cells plays an important role for the recruitment of CD4+ T-cells into the brain.

## Discussion

MS is a devastating autoinflammatory disease of the CNS, characterized by focal highly inflammatory plaques in the CNS. The recruitment of peripheral Th17 cells has been shown to contribute markedly to initiation of local inflammation and tissue destruction (2–6). Invasion of Th17 cells into the CNS is highly dependent on the interactions of VLA-4 expressed on T-cells with endothelial VCAM-1. Therefore, VLA-4 blocking (e.g. by Natalizumab) interferes with Th17 cell recruitment to the CNS resulting in significantly decreased local inflammation and progression of the disease (8). However, patients may experience relapses during Natalizumab therapy (10), indicating a VLA-4 independent recruitment mechanism of Th17 cell into the brain. We and others have previously proposed that MCAM, which represents a receptor of so far unknown function on T-cells, substantially contributes to brain inflammation in MS by promoting T-cell recruitment into the CNS (23, 31, 41). MCAM is expressed by central- (T_CM_) and effector memory (T_EM_) T-cells (34, 51) which represent different stages of T-cell differentiation and activation, we therefore decided to investigate both cell populations separately. However, both populations showed similar MCAM-mediated adhesion and signaling behavior in our experiments. Importantly, MCAM expression can be induced by pro-inflammatory cytokines such as TNFα and IL1α (52) and is associated with the Th17 subtype (37, 40). Moreover, the most relevant MCAM ligands, MCAM itself and laminin-411 have been shown to be expressed in both the BBB and particularly the BCSFB. Further, laminin-411 has been shown to facilitate T-cell migration into the CNS (31) *in vivo* and administration of MCAM blocking antibodies delays the disease progression of experimental autoimmune encephalitis (EAE) in WT mice (31) and the disease onset in T-cell specific VLA-4 KO mice (23).

Although MCAM-mediated T-cell adhesion has been proposed before, none of the previous studies quantified the adhesive properties of MCAM under physiological shear stress conditions. Using primary human MCAM expressing T-cells, we here show that binding of MCAM to either MCAM or laminin-411 induces a dose-dependent adhesion in a standardized *in vitro* flow chamber assay. Importantly, adhesion to MCAM was absent in MCAM- T-cells and abrogated when MCAM+ T-cells were pre-treated with a blocking anti-MCAM antibody, demonstrating that MCAM directly acts as an adhesion molecule. MCAM- cells were shown to adhere to laminin 411, probably due to the expression of integrin α6. Adding the β1-integrin ligand VCAM-1 to MCAM/laminin-411 resulted in a significantly higher T-cell adherence. Our flow chamber assays were performed in a standardized way regarding temperature, applied shear stress, cell dilution as well as quantified area and time of flow rather than representing the physiologic conditions of the cerebral microcirculation. The limited abundance of MCAM+ T-cells in the blood of healthy donors remains a technical issue, however, these data together with our biochemistry data and reporter antibody experiments demonstrate mechanistically that MCAM engagement induces the activation of intracellular signaling pathways that lead to β1-integrin activation. Interestingly, we only observed this for low concentrations of VCAM-1, indicating that the MCAM-mediated increase of cellular adhesion to VCAM-1 is of particular importance if VCAM-1 is expressed at low levels, e.g. in non-inflamed tissue or under immune suppression.

MCAM-mediated intracellular signaling *via* the SRC kinase FYN and the tyrosine kinases PLCγ, PYK2 and FAK1 have been described before in endothelial cell lines (32, 33), but not in leukocytes. We stimulated MCAM+ and MCAM- memory T-cells with MCAM or laminin-411 under shear conditions and demonstrated that MCAM engagement induces the phosphorylation of SRC, FAK1 and PLCγ1. Importantly, induction of SRC, FAK1, and PLCγ1 phosphorylation was absent in MCAM- cells or if MCAM+ cells were pre-treated with a blocking anti-MCAM antibody. Interestingly, SRC and FAK1 are known to be involved in integrin outside-in-signaling, whereas PLCγ is involved in insight-out as well as outside-in signaling of β1-integrins (53–56). By using inhibitor pre-treated and knock-down cells in flow chamber and reporter antibody experiments, we demonstrated that PLCγ1 is involved in MCAM-mediated β1-integrin activation. Inhibition of SRC or FAK1 was not sufficient to counteract MCAM-mediated adherence, indicating that other kinases upstream of PLCγ1 play an additional role. However, our blocking and knock-down experiments show that PLCγ1 activity is crucial for MCAM-mediated integrin β1 activation. Thus, specific inhibition of PLCγ1 in patients experiencing relapses during Natalizumab therapy might be a new treatment option to reduce T-cell recruitment into the central nervous system and therefore reduce relapse episodes.

Here, we provide the first functional proof of a mechanistic link between MCAM-mediated intracellular signaling and integrin activation. Notably, besides pairing with the integrin subunit α4 (forming integrin α4β1/VLA-4) integrin subunit β1 also forms heterodimers with α1, -2, -3, - 4, -5, -6, -7, -8, -9 -10, -11, and –v which then bind to different ECM molecules and signal transmitting factors such as different species of collagen, fibronectin, vitronectin, laminin, as well as thrombospondin and osteopontin, and membrane receptors such as MadCAM-1 and VCAM-1 [reviewed in (57)]. We show that MCAM+ T-cells also express integrin α5 and α6, thus, broad activation of β1-integrin by MCAM mediated PLCγ1 signaling might be a mechanism of increased cell adherence to the ECM in various tissues. However, we did not further investigate this. As MCAM and β1-integrins are also involved in cancer metastasis (11, 58, 59), our data might provide a mechanistic link between these molecules and disease progression also in that context.

Our adoptive transfer experiments implementing specific knock down of plcγ1 in MCAM expressing myelin oligodendrocyte glycoprotein-specific T-cells (2D2 donor mice) further underline the relevance of MCAM-mediated signaling *via* plcγ1 in the context of T-cell recruitment into the brain *in vivo*, however, whether additional MCAM dependent signaling pathways are involved in VLA-4 independent T-cell recruitment to the CNS and which impact MCAM mediated T-cell recruitment *via* plcγ1 activation has in the context of disease (e.g. EAE) remains to be determined. Notably, our data are line with previous studies describing impaired CNS infiltration and delay of disease onset/severity in different mouse models of MS with endothelial MCAM knock out or anti-MCAM antibody treated mice (23, 60). We have previously shown that MCAM blockade impairs the recruitment of MCAM+ T-cells to the CNS *via* the choroid plexus and delays the disease onset in EAE induced mice lacking integrin α4 expression in CD4+ T-cells and ameliorates the disease beyond the blocking of VLA-4 in a spontaneous model of MS (2D2 mice) (23).

Ours and previously published studies (23, 31, 41, 60) thus provide reliable evidence for the relevance of MCAM dependent CNS invasion of T-cells, indicating MCAM blockade to be a suitable treatment approach for MS patients. Clinical trials implementing anti-MCAM antibodies in psoriasis patients have reported limited success, however, leukocyte recruitment to the brain in MS patients might still be efficiently constrained by anti-MCAM antibody treatment.

## Conclusion

Our study using primary human T-cells in *in vitro* assays and a murine *in vivo* model of T-cell recruitment to the CNS provides first evidence for MCAM-mediated ß1-integrin activation *via* PLCγ1 engagement and subsequent amplification of CNS invasiveness.

## Data Availability Statement

The original contributions presented in the study are included in the article/**Supplementary Material**. Further inquiries can be directed to the corresponding author.

## Ethics Statement

All experiments including human material were reviewed and approved by the local ethics committee (Ethik-Kommission der Ärztekammer Westfalen-Lippe und der Medizinischen Fakultät der Westfälischen-Wilhelms-Universität) and performed according to the Declaration of Helsinki. The patients/participants provided their written informed consent to participate in this study. All experiments involving mice were reviewed and approved by the responsible animal protection authority (Landesamt für Natur- Umwelt- und Verbraucherschutz Nordrhein-Westfalen) and conducted according to the German Animal Protection Law.

## Author Contributions

LZ, SH, PK, and KK performed the experiments. LZ analyzed the data and wrote the manuscript. AZ revised the manuscript. TS-H, HW, NS, and AZ conceived and designed the study. All authors contributed to the article and approved the submitted version.

## Funding

This study was supported by the German Research Foundation (DFG) Grant CRC128 Project B1 (NS, AZ).

## Conflict of Interest

The authors declare that the research was conducted in the absence of any commercial or financial relationships that could be construed as a potential conflict of interest.
