# Supplementary material for: MCAM/CD146 Signaling via PLCγ1 Leads to Activation of β1-Integrins in Memory T-Cells Resulting in Increased Brain Infiltration
Source: Front Immunol. 2020 Dec 14;11:599936. doi: 10.3389/fimmu.2020.599936 (PMC7767877; doi:10.3389/fimmu.2020.599936)
Supplement: Supplementary file 1 [file DataSheet_1.pdf]

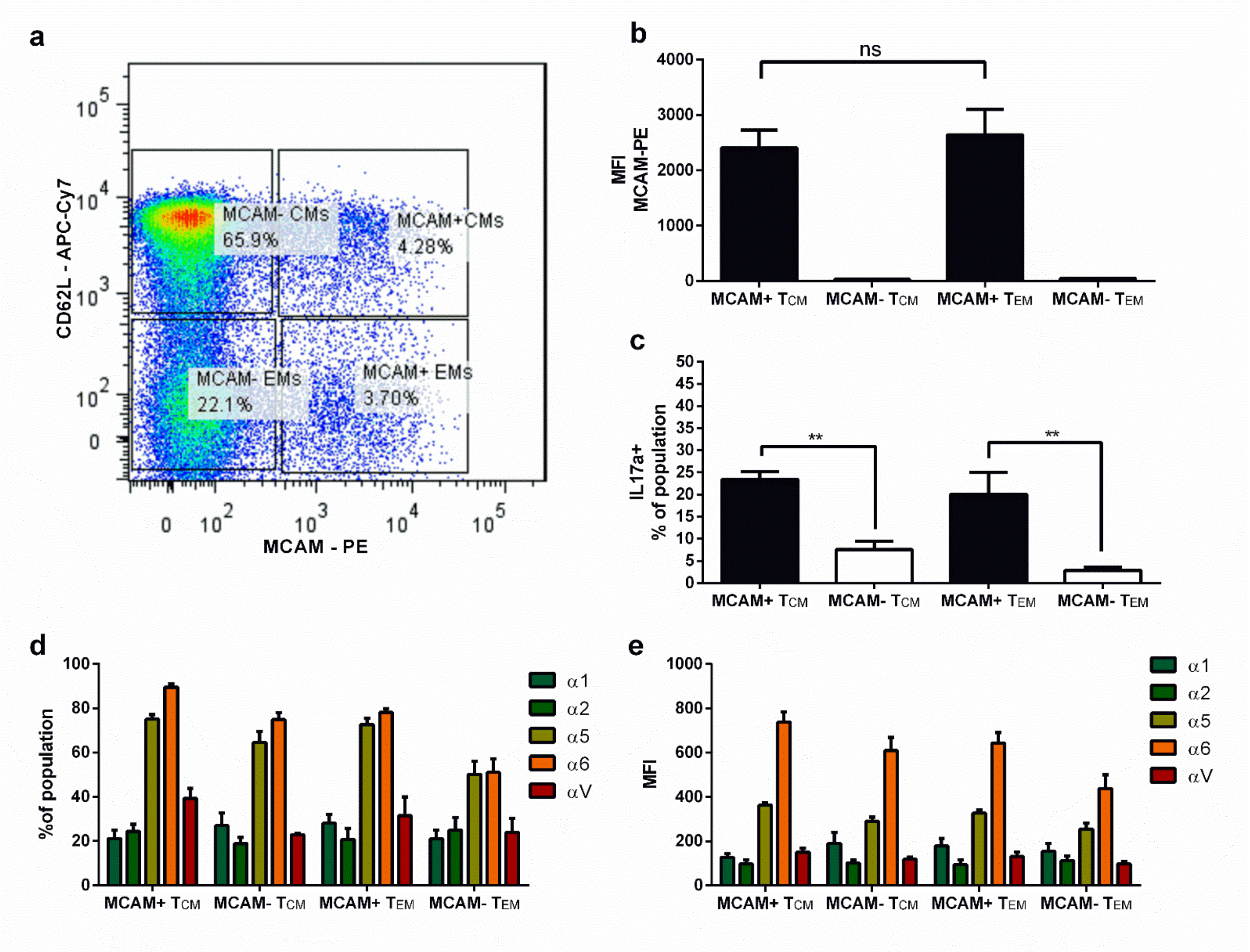

Figure S1: Human T_{CM} and T_{EM} were isolated by flourescence activated cell sorting (FACS) based on expression of CD4, CD45RA and CD62L (T_{CM} CD4+CD45RA-CD62L+; T_{EM} CD4+ CD45RA-CD62L-; **a**). The expression levels of the MCAM molecule were shown to be similar in MCAM+ T_{EM} and T_{CM} (**b**; n=4). Expression of IL-17a was associated to MCAM+ T_{EM} and T_{CM} (**c**; MCAM+ T^{CM} vs MCAM- T_{CM} p=0.0058, MCAM+ T_{EM} vs MCAM- T_{EM} p=0.0028; n=5). Flow cytometric quantification showed high expression of integrin α5 and α6 but low expression of integrin α1, α2 and αV in all four tested t-cell populations (**d**, **e**; n=5).

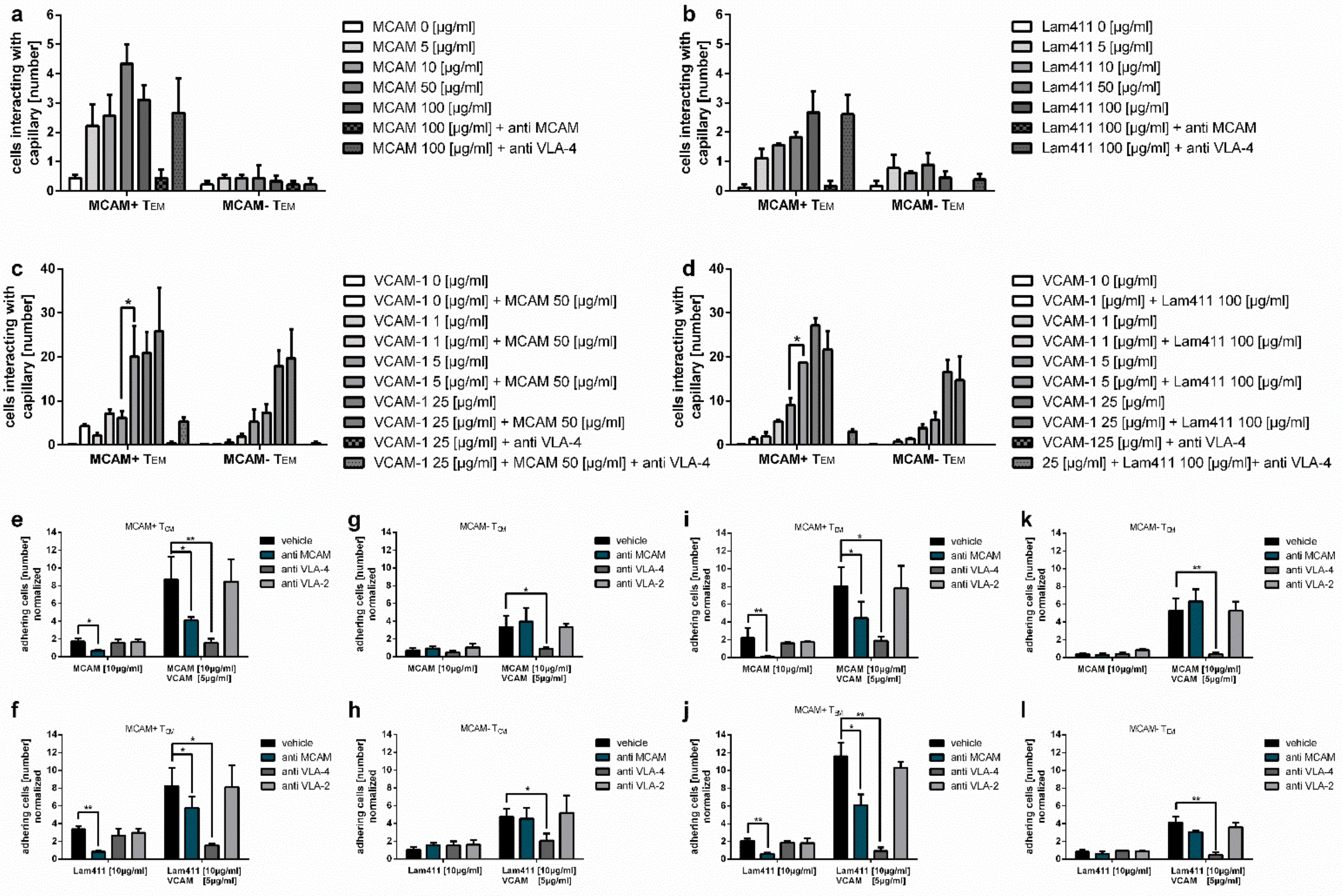

Figure S2: Along with the experiments presented in figure 1 the same flow chamber experiments were performed implementing MCAM + and - T_{EM}. Firm adherence of MCAM+ and MCAM- T_{EM} was assessed to be dependent on the substrate dose in flow chambers coated with increasing amounts of MCAM (0; 5; 10, 50, 100 µg/ml; n=3; **a**) and laminin-411 (0; 5; 10, 50, 100 µg/ml; n=3; **b**). Firm adherence of MCAM+ and MCAM- T_{EM} was assessed in flow chambers using increasing amounts of VCAM-1 (0; 1; 5; 25 µg/ml) plus constant amounts of MCAM (50 µg/ml; p=0.04; n=3; **c**) or laminin-411 (100 µg/ml; p=0.01; n=3; **d**) and found to be increased in MCAM+ T_{EM} if VCAM-1 at 5 µg/ml was co-coated with MCAM or laminin-411. To ensure the specificity of our flow chamber assays several control experiments were performed. Treatment with anti MCAM antibody before subjecting the cells to the flow chambers abolished adherence to MCAM or laminin-411 alone in MCAM expressing T_{EM}/T_{CM} (**e**, p=0.04, n=5; **f**, p=0.05, n=5; **i**, p=0.03, n=5, **j**, p=0.05, n=5). In flow chambers co-coated with either MCAM or laminin-411 and VCAM-1 pretreatment of the cells with anti-MCAM antibody lead to reduced adherence and pretreatment of the cells with Natalizumab (anti VLA-4) resulted in reduced adherence to the level of pure MCAM/laminin-411 coating in MCAM+ T_{EM} and T_{CM} (**e**, p= 0.004, n=5; **f**, p=0.01, n=5; **I**, p=0.01, n=5, **j**, p=0.00, n=5). In MCAM- T_{EM} and T_{CM} MCAM blocking had no effect on their adherence to MCAM or laminin-411 +/- VCAM-1 whereas pretreatment with Natalizumab (anti VLA-4) lead to reduced adherence (**g**, p=0.04, n=5; **h**, p=0.03, n=5; **k**, p=0.002, n=5; **l**, p=0.007, n=5). Anti VLA-2 antibody treatment did not affect T-cell adherence to MCAM/Lam441 + VCAM-1.

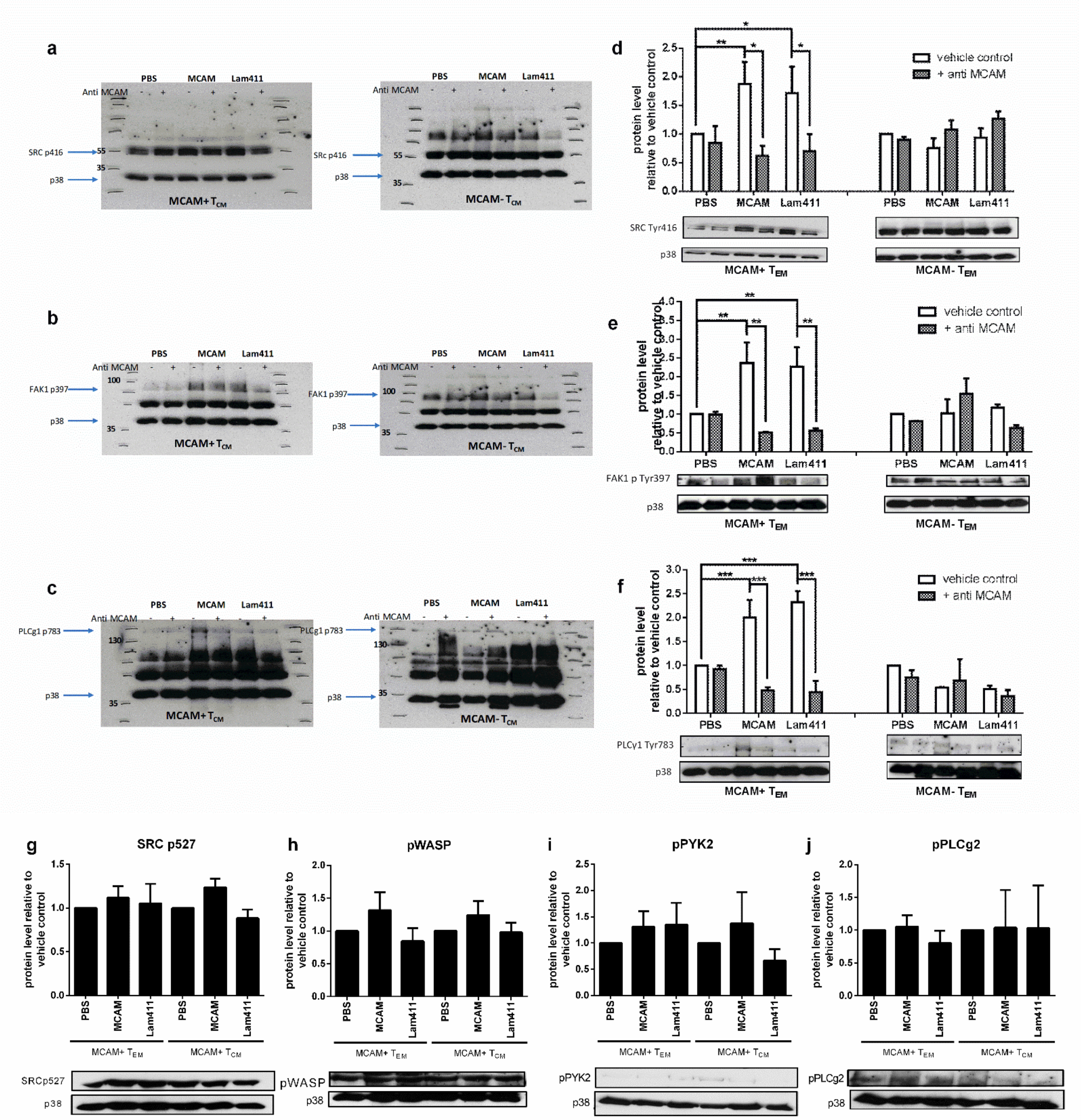

Figure S3: *In vitro* rolling experiments in MCAM and laminin-411 coated dishes were performed to trigger MCAM signaling in T_{EM} and T_{CM}. Western blotting analysis and quantification show increased phosphorylation of SRCp416 (**a**, whole membrane T_{CM}; **d**, p=0.007, p=0.01; n=4), FAK1p397 (**b**, whole membrane T_{CM}; **e**, p=0.001, p=0.009; n=3) and PLCγ1p783 (**c**, whole membrane T_{CM}; **f**, p=0.0006, p=0.0006; n=5) in MCAM+ T_{EM} and T_{CM}. This effect is abolished by application of anti-MCAM to the cells before plating them on the coated surface (**d**; T_{EM}: p=0.04, p=0.04; **e**, p=0.004, p=0.009; **f**, p<0.0001, p<0.0001). Representative blots are shown for each kinase. Western blot analysis of primary human MCAM+ T_{EM}/T_{CM} was performed upon MCAM stimulation assessing the phosphorylation of SRCp527 (**g**, n=10), PYK2p402 (**i**, n=4), Plcγ2p759 (**j**, n=3) and WASPp290 (**h**, n=9), showing no increased kinase phosphorylation.

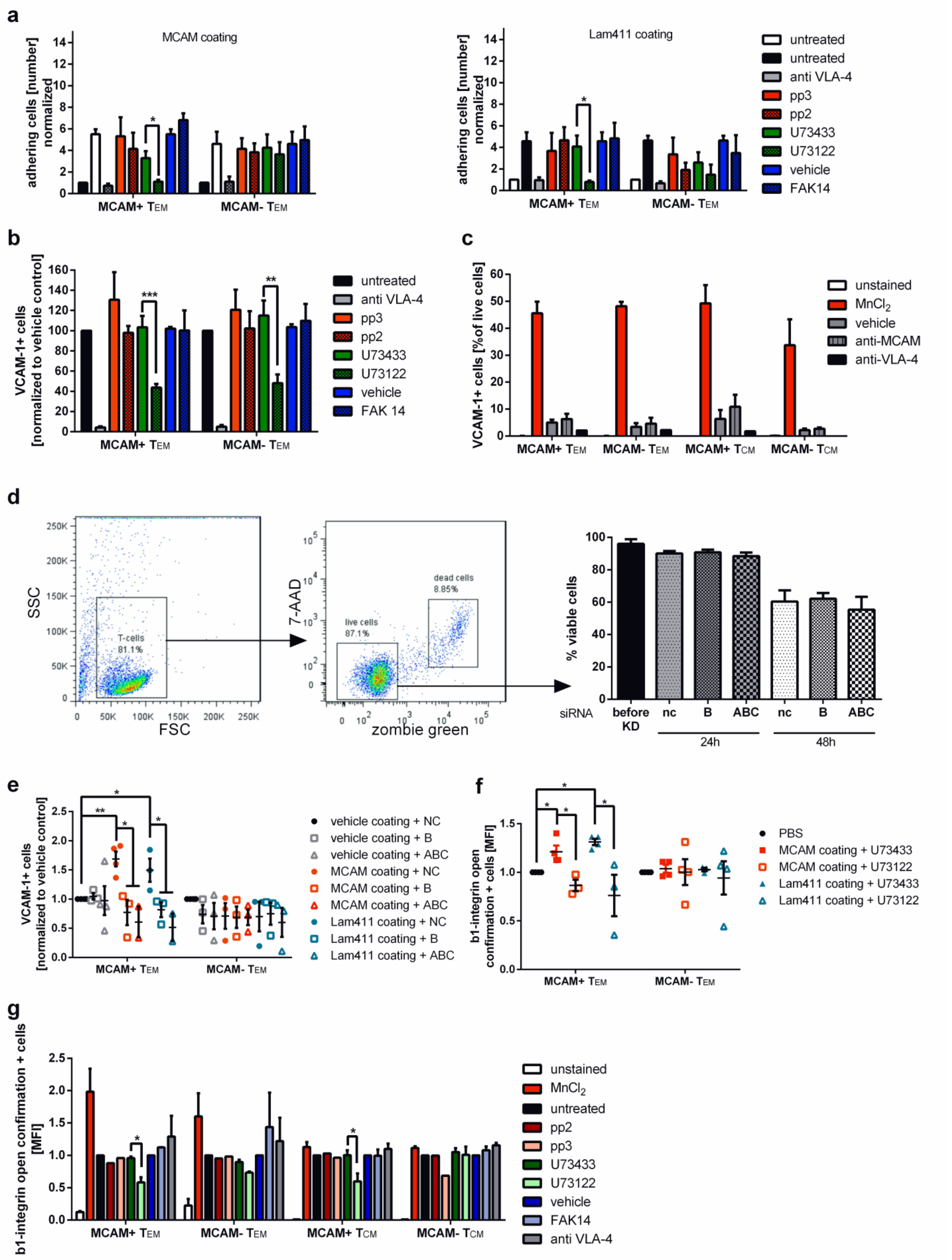

Figure S4: To confirm the involvement of SRC, PLCγ and FAK1 in MCAM mediated intracellular signaling primary human T_{EM} were pretreated with the SRC inhibitor pp2 or the respective control pp3, the PLCγ inhibitor U73122 or the respective control U73433, or the FAK1 inhibitor FAK14 before subjecting the cells to flow chamber assays. Flow chambers were coated with MCAM or laminin-411 plus VCAM-1. Only inhibition of PLCγ by U73122 resulted in

decreased adherence on MCAM + VCAM-1 or laminin-411 + VCAM-1 coated flow chambers in MCAM+ T_{EM} (**a**, p=0.01, p=0.01, n=4) and confirmed in a VCAM-1 binding assay (**b**, p=0.0006, p=0.007, n=5). Functionality of the VCAM-1 binding assays was assessed using $MnCl_2$ to induce integrin activation as a positive control, Natalizumab (anti VLA-4) as a negative control and anti MCAM as a neutral treatment control in MCAM+/- T_{EM}/T_{CM} (**c**, n=4). To specify the inhibition of PLCγ to the subtype PLCγ1, a PLCγ1 knock down using specific siRNA oligonucleotides in primary human MCAM+ T_{EM} was performed before analyzing the cells in MCAM/laminin-411 + VCAM-1 binding assays (nc: negative control, B: oligonucleotide B, ABC: mixture of oligonucleotide A, B and C). Cell viability was assessed before and after performing the knock down (24h, 48h) by a flow cytometry-based approach and shown to be comparable in all treatment groups (siRNA nc, B, ABC) and was ~ 90% 24h and ~60% 48h after performing the knock down (**d**, n=3). VCAM-1 binding assays further confirmed the induction of VCAM-1 binding upon MCAM stimulation with MCAM and laminin-411 protein as well as the involvement of PLCγ1 in MCAM mediated VCAM-1 binding (**e**, p=0.001, p=0.02, p=0.03, p=0.03, p=0.02, p=0.01, n=3-4). Both MCAM stimulation with MCAM or laminin-411 results in increased abundance of full open β_1-integrins on MCAM+ T_{EM} and can be reversed by administration of the PLCγ inhibitor U43122 as assessed by flow cytometry (**f,** p=0.02, p=0.02, p=0.04, p=0.04, n=4; **g**, p=0.02, p=0.04).

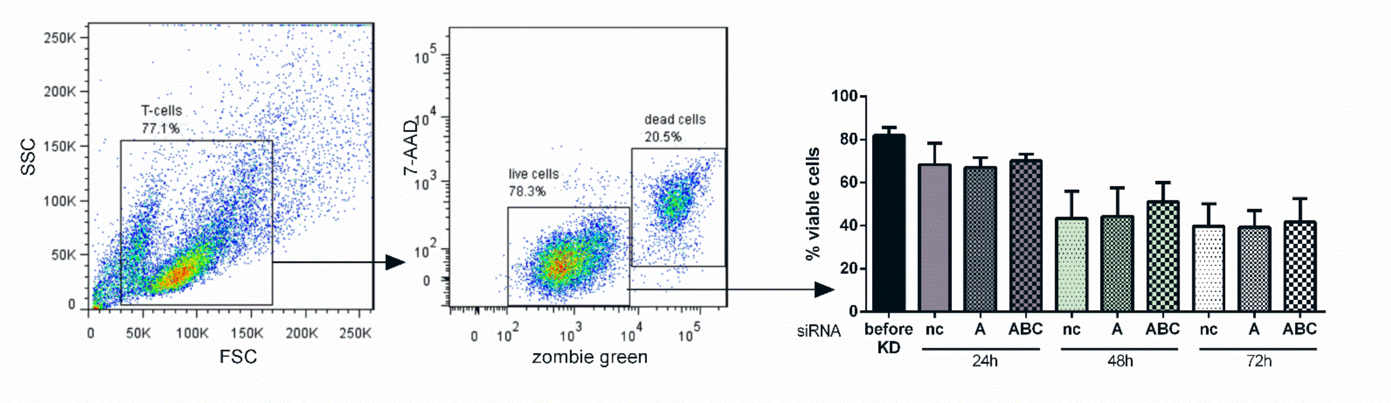

Figure S5: Murine T-cells were prepared from 2D2 mice, expanded and differentiated into MCAM+ Th17 cells *in vitro* and plcγ1 expression was knocked down by electroporation and transfer of specific siRNA oligonucleotides (nc: negative control, A: oligonucleotide A, ABC: mixture of oligonucleotide A, B and C). Cell viability was assessed before and after performing the knock down (24h, 48h, 72h) by a flow cytometry-based approach and shown to be comparable in all treatment groups (siRNA nc, A, ABC) and was ~ 70% 24h ~50% 48h and 72h after performing the knock down (n=3).
